# Automated Progress-Monitoring for Literate Language Use in Narrative Assessment (LLUNA)

**DOI:** 10.3389/fpsyg.2022.894478

**Published:** 2022-05-16

**Authors:** Carly Fox, Sharad Jones, Sandra Laing Gillam, Megan Israelsen-Augenstein, Sarah Schwartz, Ronald Bradley Gillam

**Affiliations:** ^1^Department of Data Analytics & Information Systems, Utah State University, Logan, UT, United States; ^2^Department of Communication Disorders & Deaf Education, Utah State University, Logan, UT, United States; ^3^Department of Psychology, Utah State University, Logan, UT, United States

**Keywords:** computer automation, progress-monitoring, narrative, literate language, natural language processing

## Abstract

Language sample analysis (LSA) is an important practice for providing a culturally sensitive and accurate assessment of a child's language abilities. A child's usage of literate language devices in narrative samples has been shown to be a critical target for evaluation. While automated scoring systems have begun to appear in the field, no such system exists for conducting progress-monitoring on literate language usage within narratives. The current study aimed to develop a hard-coded scoring system called the Literate Language Use in Narrative Assessment (LLUNA), to automatically evaluate six aspects of literate language in non-coded narrative transcripts. LLUNA was designed to individually score six literate language elements (e.g., coordinating and subordinating conjunctions, meta-linguistic and meta-cognitive verbs, adverbs, and elaborated noun phrases). The interrater reliability of LLUNA with an expert scorer, as well as its' reliability compared to certified undergraduate scorers was calculated using a quadratic weighted kappa (*K*_*qw*_). Results indicated that LLUNA met strong levels of interrater reliability with an expert scorer on all six elements. LLUNA also surpassed the reliability levels of certified, but non-expert scorers on four of the six elements and came close to matching reliability levels on the remaining two. LLUNA shows promise as means for automating the scoring of literate language in LSA and narrative samples for the purpose of assessment and progress-monitoring.

## 1. Introduction

Language sample analysis (LSA) is used clinically for both language assessment and progress-monitoring. It involves the elicitation of some form of connected speech from a client that is recorded, transcribed, and then systematically analyzed. Traditionally, speech language pathologists (SLPs) obtain conversational, personal, and/or fictional narratives from students for use in LSA. There are advantages and disadvantages for each type of discourse, however in general, fictional narratives may provide a more complex language sample than when a child is asked to participate in a casual conversation, or to relate a personal story (Westerveld et al., 2004). A child's ability to produce grammatical and syntactically complex narratives is an important developmental and educational milestone, as cited by both the Common Core State Standards (CCSS; “National governors association and council of chief state school officers,” 2011) and a wealth of research suggesting that poor narrative microstructure can indicate delayed or impaired language (Liles et al., 1995; Greenhalgh and Strong, 2001; Justice et al., 2006; Gillam et al., 2017).

Narrative microstructure is comprised of the words, phrases, and sentences within the discourse of a story (Hughes et al., 1997). In order to analyze a client's use of narrative microstructure, a language sample can be coded with a microstructure rubric (Justice et al., 2006). There are several research-validated tools available to clinicians and educators to choose from including the Index of Narrative Microstructure (INMIS), Sampling Utterances and Grammatical Analysis Revised (SUGAR), Narrative Scoring Scheme (NSS) and Monitoring Indicators of Scholarly Language (MISL), among others (Justice et al., 2006; Heilmann et al., 2010a; Gillam et al., 2017; Pavelko and Owens, 2019). Microstructure covers a wide range of indices, such as language productivity, lexical diversity, syntactic complexity, grammaticality, and fluency, among others. The current study focused specifically on a variety of microstructure measures known as literate language. A quality story contains complex and elaborated sentences, which are constructed through the usage of coordinated and subordinated conjunctions joining clauses, meta-linguistic and meta-cognitive verbs, adverbs, and elaborated noun-phrases (Westby, 2005). These microstructure conventions are collectively referred to as literate language since they are critical to not only narrative, but general academic discourse encountered both in the classroom and in text. Children who struggle to incorporate such syntactically complex language not only perform lower on narrative production and comprehension tasks but are at greater risk for poor academic performance (Bishop and Edmundson, 1987; Wetherell et al., 2007). Prior work has shown that children with language impairments tend to incorporate fewer of these literate language conventions than their typically developing peers (Greenhalgh and Strong, 2001), making narrative microstructure a critical assessment, progress-monitoring, and intervention target for clinicians and educators.

Potentially the biggest drawback to any microstructure rubric used as a part of LSA is the amount of time and resources required. Most of these instruments require that the user conduct the analysis by hand, while further relying on the transcription and hand-coding of narratives, which requires some training and practice in order to score reliably. It is these factors that reduce the likelihood that clinicians or educators will employ LSA as a part of their typical assessment protocol, even though it is the gold-standard for both assessment and progress monitoring (Tager-Flusberg and Cooper, 1999; Heilmann et al., 2010c). In their 2016 report, Pavelko et al. surveyed approximately 1,400 SLPs, and found that only 2/3 had used LSA at least once in the past academic year (Pavelko et al., 2016), with more than 50% having used LSA less than ten times. In addition, LSA was most commonly utilized for conversational samples, not narratives. These findings are not surprising however, given that SLPs have consistently reported time-constraints as the greatest barrier to conducting LSA (Westerveld and Claessen, 2014; Pavelko et al., 2016; Fulcher-Rood et al., 2018).

The Systematic Analysis of Language Transcripts (SALT) (Miller and Chapman, 2004) and the Child Language Analysis System (CLAN) (MacWhinney and Snow, 1991) both offer a means of reducing the time spent in LSA, by automatically generating language indices of interest for clinicians and researchers evaluating child language. Both programs can be used to assess a wide variety of descriptive (e.g., mean length of utterance, number of different words, etc.), syntactic/morphological, semantic, fluency, and discourse measures. In addition, several helpful tutorials are available to walk the user through their usage (Finestack et al., 2020; Pezold et al., 2020). However, in order to generate these metrics, the user is required to manually insert program specific codes within the language sample transcripts, which has the potential to be time-consuming and require training to implement reliably. These requirements may at least partially contribute to the finding that of the approximately 886 SLPs (66% of the original sample) who reported using LSA in the Pavelko et al. (2016) survey study, only 29% reported using a specific LSA method/protocol and of those, only 24% or about 62 SLPs, reported using SALT (with even fewer using CLAN). Meaning that while helpful computer-aided analysis systems exist, evidence suggests that they are not widely implemented by clinicians.

The use of LSA has seemingly fallen into what is known as the “research-to-practice gap”, described as the disconnect between what is considered best practice based on empirical evidence, and what is feasible for clinicians and educators to implement (Olswang and Prelock, 2015). The question therefore becomes how to reconcile the barriers faced by clinicians against the need to use best practices.

Natural language processing (NLP) offers a potential solution to reducing the time spent conducting the analysis component of LSA, such that no manual coding is required. Previous applications of NLP within the domain of child language assessment include usage for diagnosis, such as the prediction of language impairment from children's transcribed language (Gabani, 2009; Hassanali et al., 2012) and in the automated analysis of language conventions within language samples (Hassanali et al., 2014). For example, Gabani (2009) examined the predictive accuracy of several machine learning models in determining language impairment (LI) status in adolescent children (13–16 years) trained upon linguistic features which were extracted from their narrative language samples. The training corpus was comprised of narrative retells and personal narratives collected from adolescents with typical language (*n* = 99) and with LI (*n* = 19). Machine learning models were trained on a number of linguistic features, which were extracted using NLP methods, including language productivity (e.g., MLU, NTW), morphosyntactic skill (e.g., subject-verb agreement), vocabulary (e.g., NDW), fluency (e.g., repetitions and revisions), and perplexity values (i.e., inverse probability of particular part-of-speech combinations amongst a set of words). Cross-validated results indicated that machine learning models achieved modest accuracy levels for both the retell and personal narrative conditions, with a F1 score of 72.22 and 56.25%, respectively.

These results were improved upon by Hassanali et al. (2012) with the addition of features generated through Coh-Metrix, an open-source text analysis tool. These additional features included: readability, situation model features (e.g., causal features, temporal features), word features (e.g., frequency of content words), syntactic features (e.g., use of connectives, number of noun phrases), and referential features (e.g., number of adjacent utterances with argument overlap). The highest performing model achieved an F1 score of 91.4% for narrative retell and 66.7% for personal narratives. While classification accuracy of LI status based on linguistic features from personal narratives remained low, Hassanali et al. (2012) found higher accuracy for models trained on features extracted from retells. These results provide evidence for the utility of NLP in extracting relevant linguistic features from narrative samples that could be used to help clinicians expedite the screening process for language impairment in children.

Beyond diagnosis, efforts have been made to automate particular language assessment tools, such as the index of productive syntax (IPSyn) (Scarborough, 1990). The IPSyn is designed to measure the development of critical syntactic forms in expressive language. The IPSyn includes 60 items split across four main syntactic constructs: noun phrases, verb phrases, questions and negations, and sentences. All items are scored based on their number of unique instances. Several automated scoring systems exist for the IPSyn, however, the automatic computation of the IPSyn system (AC-IPSyn) has shown the highest levels of accuracy (Hassanali et al., 2014). The AC-IPSyn incorporates two common NLP methods including part-of-speech (POS) tagging and syntactic parsing, in combination with hard-coded linguistic rulesets that are used to identify the 60 IPsyn structures and output the associated scores. AC-IPSyn was found to have a point-by-point accuracy of 96.9 and 96.4% when evaluated on two datasets, one which included 20 transcripts elicited from young (2–3 years) typically developing (TD) children and one which included 20 transcripts from early school-age (6 years) children (TD = 10, LI = 10). The authors concluded that use of this automated assessment tool could significantly cut down on the time spent in analysis while still maintaining high levels of accuracy.

Each of these studies have focused on either automation of diagnosis/screening or language assessment, both of which are typically done prior to and post intervention. LSA is also often used in progress-monitoring however, to track changes in language skills throughout intervention. Progress-monitoring tools are designed to provide insight into changes in language skill, rather than provide a comprehensive assessment of language ability. To our knowledge, limited options are available for automatically analyzing language measures for progress-monitoring purposes. In addition, we are not aware of an automated microstructure assessment tool that includes measures of literate language, even though such measures could be of interest to clinicians working with school-age clients (Bishop and Edmundson, 1987; Wetherell et al., 2007). There remains a gap then, in the development of a code-free automated assessment tool that is designed for (1) progress-monitoring that (2) calculates measures of literate language.

The primary aim of the current study was to address this gap by developing an automated progress-monitoring system for evaluating literate language use in narrative assessment (LLUNA) based on an existing tool called the Monitoring Indicators of Scholarly Language (MISL) (Gillam et al., 2017). While the MISL can be used as a narrative assessment tool, it has also been validated for usage in tracking the development of children's narrative language abilities throughout intervention, providing SLPs with valuable insights into the progress of their students/clients. Automation of the MISL would allow for more frequent usage of this tool by SLPs, as it would reduce the time spent conducting the analysis.

A secondary aim of this study was to determine the clinical utility of LLUNA by determining whether the accuracy of its generated scores was on par with manually-produced scores. It is recommended that individuals who want to utilize the MISL rubric complete a certification course and achieve a minimum of 85% reliability with a gold-standard expert. However, even after achieving certification, measurement error due to rater drift, fatigue, and other sources, may impact the accuracy of scores produced by the average, trained rater. In order for the LLUNA system to be clinically useful, its generated scores would need to be as reliable as scores produced manually by a trained, but non-expert scorer. Therefore, the accuracy levels achieved by LLUNA were compared against the interrater reliability of trained, but non-expert MISL scorers with an expert scorer. These aims were addressed with the following research questions:

1. What level of scoring accuracy is achieved by LLUNA on each measure of the MISL, as determined by the gold-standard expert scores?
2. Does LLUNA match the level of interrater reliability achieved by trained, non-expert scorers with gold-standard expert scores?

## 2. Methodology

### 2.1. Language Samples

The accuracy of LLUNA was evaluated on a corpus of 50 oral narratives randomly selected from a prior study, which included a normative sample of 414 English-speaking children aged 5;0–9;11 years. All narratives were elicited in response to the *Alien Story* subtest of the Test of Narrative Language (Gillam and Pearson, 2004). This subtask uses a single-scene picture prompt to elicit a semi-spontaneous narrative language sample. Stories ranged in length between 1 and 41 utterances, as measured in communication-units (*M* = 13.2, *SD* = 7.6), and between 1 and 3 min in length (*M* = 1.05, *SD* = 0.62). Of note, some of the included narratives were quite short in length, with 16% shorter than nine utterances in length, and 40% shorter than 1 min in length. While several studies have indicated that narratives as short as nine utterances or 1 min in length can still provide accurate representations of children's language abilities (Heilmann et al., 2010b), we chose to include narratives that fell below these minimums. Mainly, we wanted to ensure that the full range of MISL scores were represented within our narrative samples, including scores of zero. Since measures were on the microstructure level, the representation of language samples for which measures like coordinating conjunctions (e.g., “and”) had a score of zero necessitated the inclusion of some very short narratives. The sample of 50 narratives evaluated in the current study were thus able to cover the range of scores (0–3) for each of the six microstructure elements.

Narratives were digitally recorded and transcribed using Systematic Analysis of Language Transcription (SALT) software, according to the conventions outlined in Miller and Chapman (Miller and Chapman, 2004). Two research assistants who were blind to the purpose of the study independently transcribed the audio verbatim. Reliability was assessed by independently double-transcribing a random selection of 20% of the transcripts, which averaged at 96% agreement across words transcribed, mazing, and morpheme segmentation.

Prior to analysis, each individual transcript within the corpus was read into R and cleaned of all unwanted characters, which included mainly SALT annotations. In addition to cleaning annotations, all words within mazed utterances were dropped from the transcripts to exclude repetitions, false-starts, revisions, and other disfluencies from analysis. Disfluencies were excluded as they were not relevant in the assessment of literate language usage. No alteration or correction was made to grammatical errors present within the main (non-mazed) transcript texts, as ungrammatical words were not coded for in the transcripts. Cleaned transcripts were converted into individual strings and paired with a unique identifier to be later matched with their corresponding MISL scores.

### 2.2. MISL Measures

The LLUNA system was designed to match the microstructure subsection of the MISL, which is a progress-monitoring rubric designed to evaluate school-age children's narrative language; narrative macrostructure was evaluated in a separate study (Jones et al., 2019). A total of six literate language elements are measured in the microstructure subsection, including coordinating and subordinating conjunctions, meta-cognitive and meta-linguistic verbs, adverbs, and elaborated noun phrases. Five of the six literate language elements of the MISL are scored on scale of 0–3 based on the number of unique instances of words that fall within the given category (e.g., “and” and “then” for coordinating conjunction), where zero indicates no instances and three indicates three or more. The remaining element, elaborated noun phrase (ENP), is scored on a scale of 0–3 based on the number of modifiers that precede a noun in a noun phrase, where zero indicates a noun in isolation and three indicates three or more modifiers preceding a noun (e.g., the big green frog). See Table 1 for full list of elements and definitions. These measures are well-suited to computer automation because the scoring conventions are objective and could be operationalized into hard-coded rulesets. This also meant the LLUNA system could be designed and then evaluated on a small sample of observations, since no data was needed to train a statistical learning model.

**Table 1:** Literate language measures defined.

**Term**	**Definition**
Coordinating conjunction	Words that connect two independent clauses, such as *and, but* and *or*.
Subordinating conjunction	Words that connect an independent and dependent clause, such as *because, therefore*, or *when*.
Meta-linguistic verbs	Verbs that indicate the act of dialogue, such as *yelled or said*.
Meta-cognitive verbs	Verbs that indicate thoughts, feelings, and character perspective, such as *wondered, thought*, or *decided*.
Adverbs	Words or phrases which modify the degree, time, manner or place of a verb or adjective.
Elaborated noun phrase	Noun phrases that contain a set of modifiers that elaborate on the given noun, e.g., *The big black dog*.

### 2.3. Manually-Produced Scores

#### 2.3.1. Gold-Standard, Expert Scores

To determine the accuracy of scores generated by LLUNA, they were compared to the gold-standard scores produced by an expert, certified SLP with 5 years of MISL evaluation experience. This expert produced scores for all 50 narratives included in the sample, but was not involved in the development of the LLUNA scoring system. All expert scores were compiled in a comma separated value (.csv) file, imported into R, and assigned to their corresponding cleaned narrative by matching their unique identification number. The resulting dataframe constituted of a unique identifier, the associated narrative string and the individual expert scores for each of the six microstructure elements.

#### 2.3.2. Non-expert Scores

All language samples were also manually scored by a small team of four non-expert scorers who were undergraduate or masters students studying speech-language pathology. Scorers had previously received certification training on how to identify literate language conventions within narrative language samples and were required to have achieved at least 85% point-by-point interrater reliability with the expert scorer across five language samples. A comparison of the expert scores to both LLUNA and non-expert produced scores served as a means of establishing how reliably LLUNA could generate scores as compared to a trained, but non-expert scorer who would be more representative of a typical SLP. One hundred percent of the transcripts were independently doubled-scored by a second non-expert scorer, and point-by-point interrater reliability averaged 87% across all six elements; discrepancies were resolved through consensus. All non-expert MISL scores were added to the dataframe based on the language sample identifier.

### 2.4. LLUNA Development

A separate hard-coded function was designed in the R statistical environment to automatically score each of the six literate language elements included in MISL microstructure subsection. The LLUNA functions utilized several components for scoring, in different combinations, including: predefined word-banks, parsing and string manipulation, part-of-speech (POS) tagging, and a scoring scheme. The rulesets for subordinating conjunctions, meta-cognitive verbs, and meta-linguistic verbs involved the fewest number of components, mainly because these elements are the least susceptible to semantic ambiguity (i.e., varying class/meaning of a word depending on its usage within a given context). Given the straightforward nature of identifying these elements, the scoring functions were developed by first creating a word-bank object of common, age-appropriate examples from each element (e.g., laughed was included under meta-linguistic verbs, chortled was not). Next, each function was written to import the clean transcript, parse the text into separate word strings, and then use string detection to match words within the transcript to those included in the associated word-bank. The number of unique matches was summed and a series of if:else statements were used to output the appropriate score (i.e., zero for no matches, one for one unique match, and so on) that was capped at 3 to match the MISL rubric.

The function for the fourth microstructure element, coordinating conjunctions, was developed in a similar manner, but required an additional component. As with the previously mentioned elements, a word-bank was compiled to contain typical, age-appropriate coordinating conjunctions. These include *for, and, nor, but, or, yet*, and *so*. Next, the identification function was designed to read in the text, parse the text to word strings, and sum the number of unique matches between the text and the coordinating conjunction word-bank. An additional rule was added to the identification system to deal with habitual openers. A habitual opener describes the repeated use of a conjunction, such as *and*, to begin clauses when it serves no connective purpose and is done instead out of habit or as a filler word. This is common in children's language but should not be counted toward the usage of coordinating conjunctions. A rule was therefore included in the identification function to skip matches if three consecutive clauses began with the same coordinating conjunction's (e.g., I went to the store. *And* then I bought groceries. *And* then I went home. *And* I put the groceries away).

The rulesets for the final elements, adverbs and ENP included a dependency on the *OpenNLP* package and its POS tagging function (Hornick, v. 0.2-7). The POS function was implemented to reduce scoring error by automatically classifying the part-of-speech for semantically ambiguous words. Adverbs cover a wide variety of words, but many of these words are shared across different classes (e.g., *like* can be a preposition, adverb, conjunction, noun, verb, or adjective) and determining the word-class depends on the context it is used within. The incorporation of automated POS-tagging served as potential solution to properly identifying true instances of adverbs within the language samples. The OpenNLP POS tagger utilizes a machine learning model pretrained on a large corpus of annotated texts. This model is able to predict the most likely word class (i.e., part-of-speech) of all words within a new text, with the caveat that its predictions are more accurate on texts most similar to those it was trained on. The OpenNLP POS tagger was trained on a large corpus of newspaper articles, so it was expected that POS-tagging within LLUNA on children's narrative transcripts would produce error. It was also expected, however, that a POS tagger would be more efficient and accurate than manually POS-tagging individual words (out of context). POS tagging was added to the adverb identification function, such that the transcript was read in, parsed to word strings, POS tagged, and then the number of unique words tagged as adverbs were summed and assigned the appropriate score (0–3).

The ruleset for ENP also utilized POS tagging to convert the words within each narrative transcript to the corresponding part-of-speech (e.g., *the quick girl ran*→*determiner adjective noun verb*). ENP is scored based on the number of modifiers that precede a noun. Modifiers were limited to determiners, numbers, pronouns, adverbs, and adjectives. In addition, in grammatical speech these modifiers occur in certain orders, for example, a determiner should not follow an adjective within the same noun phrase (e.g., *silly the girl* is not grammatical). Non-grammatical uses of ENP were not counted toward the final score. With these constraints in mind, a scoring scheme was designed in place of a word-bank to contain all possible permutations of POS combinations (given the selected modifiers) that could precede a noun and result in a score of a 0 (e.g., noun in isolate) to 3 (e.g., three or more modifiers preceding a noun). Thus, the ENP identification function first read in the text, parsed the text to word strings, converted all words to POS tags, and then identified the longest combinations of POS-tags within the text that matched the scoring scheme, before assigning the appropriate score.

Each of the six element identification functions were built into a for-loop, allowing for the entire corpus to be scored at the same time. Each set of scores (*N* = 50) were concatenated to the existing data frame containing the language sample ID, expert scores, and non-expert scores for each literate language element, such that comparisons could be made across each source (expert, non-expert, LLUNA) of MISL scores. An example of the LLUNA output can be found in the Supplementary Materials.

### 2.5. Assessment of Interrater Reliability

Once all scores were compiled, the accuracy of LLUNA-generated scores and their reliability levels as compared to non-expert scores were evaluated using a quadratic weighted kappa (*K*_*qw*_). *K*_*qw*_ is commonly used for analyzing the accuracy and interrater reliability of automated scoring systems (Dikli, 2006), as it is a recommended metric for ordinal classification problems (Ben-David, 2008). *K*_*qw*_ has several advantages over other classification metrics in that it can both weight the probability of chance agreement between raters and differentially weight the distance of disagreement between raters (Cohen, 1968). This means that a difference of one point between scores is not weighted as heavily as a difference of two or three points. The possible values of K_*qw*_ range between 0 (meaning no scores overlap) to 1 (meaning perfect overlap between scores). Within the automated scoring literature, a *K*_*qw*_ of 0.60 or above is considered a “good” level interrater reliability (Dikli, 2006). However, as with most interpretations of effect sizes, this threshold should ideally be problem dependent. In order to add greater validity to our analyses, K_*qw*_ for both the LLUNA-generated and non-expert scorers was calculated against the gold-standard expert scores. This allowed us to both evaluate the accuracy of the LLUNA system, while also providing insight into how its levels of interrater reliability compared to that of trained, but non-expert scorers.

## 3. Results

Research question one aimed to determine the accuracy of the LLUNA-generated scores across the six measures, as determined by the gold-standard expert scores. Thus, the first set of calculations evaluated the K_*qw*_ between the LLUNA-generated and expert scores for each of the six elements, see Table 2. Results indicated that the LLUNA-generated scores for each of the six measures achieved a *K*_*qw*_ of 0.60 or above, with kappa values ranging between 0.74 and 0.89. LLUNA-generated scores for meta-cognitive and meta-linguistic verbs both had the highest levels of accuracy, *K*_*qw*_ = 0.89. The lowest K_*qw*_ was observed for ENP scores, *K*_*qw*_ = 0.74, which still surpassed the threshold for “good” interrater reliability at 0.60.

**Table 2:** *K*_*qw*_ for LLUNA and expert scores.

**Microstructure element**	***K*_*qw*_**
Coordinating conjunction	0.78
Subordinating conjunction	0.88
Meta-linguistic verbs	0.89
Meta-cognitive verbs	0.89
Adverbs	0.79
Elaborated noun phrase	0.74

A second set of calculations were completed to address research question two, which aimed to investigate how the interrater reliability between LLUNA and the expert scores compared to that achieved by trained, but non-expert scorers. The *K*_*qw*_ between the non-expert and expert scores across the six elements are shown in Table 3. Results indicated that the *K*_*qw*_ between non-expert and expert scores ranged from *K*_*qw*_ = 0.52–0.86. In this case, non-experts had the highest level of accuracy in scoring coordinating conjunctions *K*_*qw*_ = 0.86, while they were least accurate in scoring adverbs, *K*_*qw*_ = 0.52. Non-expert scores on adverbs had the only *K*_*qw*_ value that was below the threshold value of 0.60, indicating low levels of interrater reliability with the expert scores. A comparison of the interrater reliability levels achieved by LLUNA and non-experts with the expert scores indicated that the LLUNA system was more accurate in scoring four of the six elements, including: subordinating conjunctions, meta-linguistic and meta-cognitive verbs, and adverbs. See Table 4. By contrast, the non-expert scorers were more accurate in scoring coordinating conjunctions and ENP. While LLUNA fell below the interrater reliability levels of non-expert scorers for these two elements, overall, the system still achieved good levels of reliability with gold-standard expert scores, with all kappa levels surpassing 0.60.

**Table 3:** *K*_*qw*_ for hand-scores (expert and non-expert).

**Microstructure element**	***K*_*qw*_**
Coordinating conjunction	0.86
Subordinating conjunction	0.71
Meta-linguistic verbs	0.83
Meta-cognitive verbs	0.75
Adverbs	0.52
Elaborated noun phrase	0.78

**Table 4:** *K*_*qw*_ for hand-scores against LLUNA.

**Microstructure element**	**Hand-scores**	**LLUNA**
Coordinating conjunction	0.86	0.78
Subordinating conjunction	0.71	0.88
Meta-linguistic verbs	0.83	0.89
Meta-cognitive verbs	0.75	0.89
Adverbs	0.52	0.79
Elaborated noun phrase	0.78	0.74

**Figure 1:**
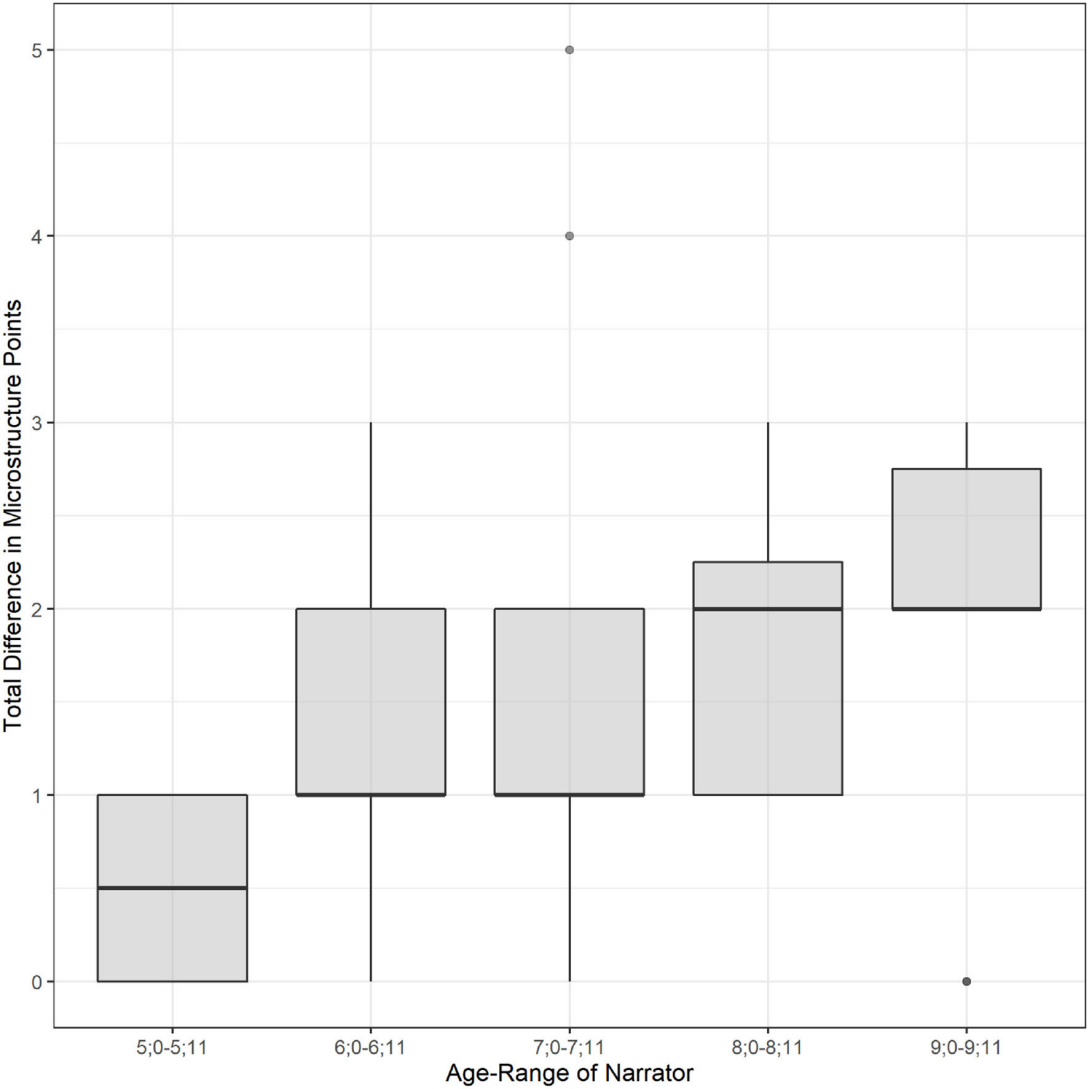
Absolute difference in total Microstructure score (Expert to LLUNA) across age-ranges (5;0-9;11).

An error analysis was conducted to investigate the potential impact of age on LLUNA scoring error. This was examined by computing the absolute difference between LLUNA and the expert across all six measures, to create a total difference score. Figure 1 indicated a modest trend, where narratives scored by LLUNA from within the older age-range (8;0–9;11) had a median total error that was one point (out of 18 total points) higher than narratives from the younger age-range (5;0–7;11).

## 4. Discussion

The purpose of the current study was to assess the feasibility of designing a computer-automated progress-monitoring system, referred to as LLUNA, that could reliably generate six measures of literate language from transcribed narrative language samples. We first aimed to determine the accuracy with which LLUNA could score each measure (coordinating and subordinating conjunctions, meta-cognitive and meta-linguistic verbs, adverbs, ENP), as determined by comparison to gold-standard expert scores. *K*_*qw*_ was calculated between LLUNA generated and expert-scores across all six measures and revealed a strong level of interrater reliability, with kappa values ranging between 0.74 and 0.89. LLUNA generated scores were most accurate for meta-cognitive and meta-linguistic verbs, while they were least accurate for ENP.

These results were not unexpected, given that the variety of meta-cognitive and meta-linguistic verbs utilized by school-age children (5;0-9;11) were limited and unlikely to be semantically ambiguous (e.g., *said* is always a meta-linguistic verb). A common case that did cause issue for LLUNA was the word “like”, which was often used as a meta-linguistic verb (e.g., and he was *like*, “Oh no, Aliens”) and was counted as a meta-linguistic verb by the expert scorer. The word “like” poses issues to the current design of LLUNA, since it is very semantically ambiguous and prone to misclassification even with the usage of a POS tagger. For now, this word will not be counted by LLUNA toward the score for meta-linguistic verbs and would need to be manually corrected by the user.

ENP was the least reliably scored by LLUNA, with a kappa value of 0.74. While this value fell above the literate cited kappa threshold of 0.60 for good interrater reliability, it was still lower than we would have liked. The ENP scorer relied on POS tagging in order to identify the longest string of modifiers preceding a noun in a noun phrase. Given that the POS tagger utilized was not pretrained on narrative samples nor language produced by children, it was likely that there would be a degree of error present within the POS tags. This error would then be translated to LLUNA's scoring of ENP. As POS tagging technology improves, we can expect the accuracy of scores generated by LLUNA to improve as well. At this point in time, however, we would recommend users do a spot-check to ensure ENP has been properly scored, in order to ensure the highest level of accuracy.

The second research question asked whether the levels of interrater reliability achieved between LLUNA and gold-standard expert scores were on par with the levels achieved by trained, non-expert scores. This second set of calculations was used to determine the clinical utility of LLUNA for progress-monitoring, such that we could speak to the scoring reliability of LLUNA scores in contrast to scores produced manually by non-expert individuals with training in MISL scoring. It was proposed that such individuals would more closely approximate the reliability levels of the average SLP with MISL training than a gold-standard expert. Result showed that LLUNA had higher levels of interrater reliability levels than non-expert scorers on four of the six literate language measures, including subordinating conjunctions, meta-cognitive and meta-linguistic verbs, and adverbs. While the difference in kappa values achieved by LLUNA and non-experts on meta-linguistic verbs was modest (*K*_*qw*_ = 0.89, *K*_*qw*_ = 0.83, respectively), the differences in interrater reliability levels were more pronounced for subordinating conjunctions, meta-cognitive verbs, and adverbs, with LLUNA having kappa values between 0.14 and 0.27 points higher than non-expert scores. Non-experts appeared to be particularly unreliable in scoring adverbs, highlighting the utility of LLUNA for increasing the reliability and efficiency of scoring for certain measures.

For the additional two measures (coordinating conjunction and ENP), LLUNA had lower *K*_*qw*_ values than the non-expert scorers, with the difference ranging from 0.04 to 0.08. These differences in reliability levels are modest but should still be taken into consideration by users of LLUNA. Scores generated by LLUNA for coordinating conjunctions and ENP could be spot-checked by either the SLP or preferably a SLP-assistant to ensure accuracy, but it is currently unknown whether this practice would be more efficient than manually-scoring these elements from the start. This point warrants future investigation to determine the time-costs of LLUNA, with some potential manual corrections, as compared to manual scoring.

Clinicians can, however, can be confident in the reliability of LLUNA for scoring several literate language elements, including subordinating conjunctions, meta-cognitive and meta-linguistic verbs, and adverbs within the investigated context (i.e., school-age narrative language samples), as evidenced by its high levels of interrater reliability with gold-standard expert scores. This preliminary investigation of LLUNA on 50 narratives of school-age children (5;0–9;11) provides evidence for its potential as a useful clinical tool for progress-monitoring in automatically generating several important measures of literate language.

Of note, while LLUNA may have been less reliable in scoring coordinating conjunctions and ENP than the trained, non-expert scorers, the non-experts in this study may still have more experience than the average SLP utilizing the MISL rubric. Though the non-expert scorers were intended to approximate the ability of a clinician trained in MISL scoring, SLPs report a lack of training on instruments as a current barrier to LSA (Pavelko et al., 2016). It can therefore be assumed that in many cases SLPs might not have the same level of experience in using the MISL, making them potentially less reliable scorers. Certification for the MISL can be obtained and is recommended, however, it is not a requirement to use this assessment tool. For those without MISL scoring certification, LLUNA may be the most reliable scoring option, even for coordinated conjunctions and ENP.

A basic visual error analysis was conducted to examine whether LLUNA scoring accuracy differed by age of the narrator. Though differences were modest, narratives elicited from children within the older age-range (8;0–9;11) had a median absolute total difference score that was one point higher than the younger age-range (5;0–7;11). Notably, stories elicited from children in the 5;0–5;11 age-range had the least amount of variation in LLUNA scoring error, with LLUNA producing no greater than one point (out of 18 total points) difference from the expert scorer. While this trend should be investigated across a larger number of samples to determine the impact of age on LLUNA scoring accuracy, it does provide preliminary evidence that LLUNA may perform with modestly higher accuracy on narratives elicited from early school-age children.

### 4.1. Clinical Implications

Usage of LSA as a gold-standard practice is critical to the accurate and culturally sensitive assessment of language skills in clients over time. Progress-monitoring of literate language is further important to tracking children's development of this important component of academic discourse. Unfortunately, both LSA and LSA for the purpose of progress-monitoring can be quite time-consuming, due to the numerous steps involved in the process (e.g., transcription, coding, analysis). In consequence, time-constraints are consistently listed as the number one barrier clinicians to LSA (Justice et al., 2006; Heilmann et al., 2010a; Gillam et al., 2017; Pavelko and Owens, 2019). To date, this has made the use of LSA and the associated assessment and progress-monitoring tools less practical. The use of computer automated assessment and progress-monitoring systems like LLUNA have the potential to attenuate some of these constraints by removing the time it takes to both code and score a transcript, while also requiring no additional time to become reliable in their usage. It is the aim of this work to make the use of the gold-standard practice of LSA more common by providing a practical solution to its implementation barriers. While LLUNA still requires the transcription of narrative samples, it does not require hand-coding or the use of various transcription conventions to run properly. In order to utilize this technology, clinicians will need to type their audio recordings into a plain-text file, upload the text file, and scores will then be automatically generated.

### 4.2. Limitations and Future Directions

There are several sources of potential limitation within the current study. One potential limitation is the generalizability of LLUNA to other LSA elicitation methods. LLUNA was designed and evaluated on narratives elicited in response to the Alien Story sub-task of the TNL. It is therefore at this time it is unknown whether the reliability levels reported in the current study will generalize to narratives elicited through different prompts, as their vocabulary may vary significantly from the sample used here. However, given that LLUNA was designed from hard-coded functions, meaning it is not a data-driven model, it is unlikely the reliability should be significantly different between prompts. On a similar note, the specific age range of the corpus used in this study allowed for the creation of limited word-banks for subordinating conjunctions, meta-cognitive and meta-linguistic verbs. It is therefore unknown how LLUNA performance might differ when applied to an older age group with a more robust vocabulary. Future investigations will determine the generalizability of LLUNA to alternative elicitation prompts and an older age-range. Finally, the contribution of ungrammatical verb forms to LLUNA scoring error was not investigated in the current study, meaning that children's usage of overgeneralization to irregular verb forms within their narratives may have limited LLUNA's ability to correctly identify all meta-cognitive and meta-linguistic verbs. This was likely not a common occurrence, however, given that LLUNA had the highest accuracy on these two measures. This consideration will be investigated in future versions of LLUNA to ensure the highest levels of scoring accuracy, but at this time may require spot checking on the part of the user.

In its current state, LLUNA is nearly ready for clinical utility. LLUNA was able to achieve strong levels of interrater reliability with an expert-scorer on each literate language element, although scores for coordinating conjunctions and ENP should be spot-checked at this point in time. The construction of LLUNA into a web-based applet is currently underway, which will allow for it to become publicly available in a more user-friendly format. In addition, efforts toward eliminating the manual transcription portion of the LSA process have recently be investigated by Fox et al. (in press), where it was found that automated speech recognition technology could be used to reliably generate transcripts for narrative language samples of school-age children (7;6–11;5) with developmental language disorder. As a next step, we plan to investigate the accuracy of a system combining LLUNA with automatic speech recognition, to determine the feasibility of automating the entire LSA process. It is our hope that streamlining LSA will make it a more accessible option to busy clinicians and educators.

## Data Availability Statement

The original contributions presented in the study are included in the article/Supplementary Material, further inquiries can be directed to the corresponding author/s.

## Author Contributions

CF conceptualized the study, developed LLUNA, ran all analyses, and wrote the initial draft of the manuscript. SJ consulted on the development of LLUNA, provided edits to the methods and results sections, consulted on project, design, and data analysis. SG mentored on the project and provided edits and revisions on all sections of the manuscript with a focus on the introduction and discussion. MI-A provided the expert scores and trained the non-expert scorers for the project, in addition to providing guidance on the language rule sets underlying the LLUNA development. SS mentored on the project and provided edits and revisions for the methods and results. RG provided the data used in this project, mentored on this project, and provided edits on the entirety of the manuscript with a focus on the methods and results sections. All authors contributed to the article and approved the submitted version.

## Conflict of Interest

The authors declare that the research was conducted in the absence of any commercial or financial relationships that could be construed as a potential conflict of interest.

## Publisher's Note

All claims expressed in this article are solely those of the authors and do not necessarily represent those of their affiliated organizations, or those of the publisher, the editors and the reviewers. Any product that may be evaluated in this article, or claim that may be made by its manufacturer, is not guaranteed or endorsed by the publisher.
