# Supplementary material for: Automated Progress-Monitoring for Literate Language Use in Narrative Assessment (LLUNA)
Source: Front Psychol. 2022 May 16;13:894478. doi: 10.3389/fpsyg.2022.894478 (PMC9150794; doi:10.3389/fpsyg.2022.894478)
Supplement: Supplementary file 1 [file Data_Sheet_1.PDF]

Supplementary Material

1 SUPPLEMENTARY FIGURE

```
narrative = read_file("/content/26713_Aliens.txt") #set the path to your narrative file
narrative #view narrative

there are these aliens. and they just got back from mars. they found a dog on the path
that they were going on. they got lost in the planet.  so they found a map. but they
got back. now they are back on their planet.  theyre happy.  they want to have a picnic.
these kids are walking where the aliens are.  and they get scared. they run home and
tell their mom and dad. but they think aliens arent real. so the kids take their parents
to see the aliens.  and the aliens are gone. they finished their picnic. and they went
to cave to hide because they were scared of humans.

enp_path = "/content/enp_perm.csv" #set the path to the ENP scoring file

LLUNA(narrative, enp_path) #execute LLUNA function to produce output

({'Adverbs': 3,
  'Coordinating Conjunctions': 3,
  'Elaborated Noun Phrase': 1,
  'Linguistic Verbs': 1,
  'Mental Verbs': 2,
  'Subordinating Conjunctions': 2},
 {'Adverbs': {'back', 'just', 'now'},
  'Coordinating Conjunctions': {'and', 'but', 'so'},
  'Elaborated Noun Phrase': [['a', 'picnic'], ['DT', 'NN']],
  'Linguistic Verbs': {'tell'},
  'Mental Verbs': {'think', 'want'},
  'Subordinating Conjunctions': {'because', 'that'}})
```

Figure S1. Example output of LLUNA scoring a narrative, # indicate comments in the code.
